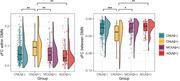# Functional connectivity alterations of the default mode network in preclinical Alzheimer's disease

**DOI:** 10.1002/alz70856_100689

**Published:** 2025-12-25

**Authors:** Woo‐Jin Cha, Dahyun Yi, Evgeny J. Chumin, Min Soo Byun, Joon Hyung Jung, Hyejin Ahn, Yu Kyeong Kim, Yun‐Sang Lee, Koung Mi Kang, Chul‐Ho Sohn, Shannon L Risacher, Olaf Sporns, Kwangsik Nho, Andrew J. Saykin, Dong Young Lee, KBASE Research Group

**Affiliations:** ^1^ Department of Neuropsychiatry, Seoul National University Hospital, Seoul, Korea, Republic of (South); ^2^ Institute of Human Behavioral Medicine, Medical Research Center, Seoul National University, Seoul, Korea, Republic of (South); ^3^ Indiana Alzheimer's Disease Research Center, Indiana University School of Medicine, Indianapolis, IN, USA; ^4^ Center for Neuroimaging, Department of Radiology and Imaging Sciences, Indiana University School of Medicine, Indianapolis, IN, USA; ^5^ Department of Psychiatry, Seoul National University College of Medicine, Seoul, Korea, Republic of (South); ^6^ Department of Psychiatry, Chungbuk National University Hospital, Cheongju, Korea, Republic of (South); ^7^ Interdisciplinary program of cognitive science, Seoul National University College of Humanities, Seoul, Korea, Republic of (South); ^8^ Department of Nuclear Medicine, SMG‐SNU Boramae Medical Center, Seoul, Korea, Republic of (South); ^9^ Department of Nuclear Medicine, Seoul National University College of Medicine, Seoul, Korea, Republic of (South); ^10^ Department of Radiology, Seoul National University Hospital, Seoul, Korea, Republic of (South); ^11^ Indiana Alzheimer's Disease Research Center, Indiana University School of Medicine, Indianapolis, IN, USA; ^12^ Department of Psychological and Brain Sciences, Indiana University, Bloomington, IN, USA; ^13^ Center for Computational Biology and Bioinformatics, Indiana University School of Medicine, Indianapolis, IN, USA; ^14^ Center for Neuroimaging, Department of Radiology and Imaging Sciences, Indiana University School of Medicine, Indianapolis, IN, USA; ^15^ Department of Medical and Molecular Genetics, Indiana University School of Medicine, Indianapolis, IN, USA; ^16^ Center for Neuroimaging, Indiana University School of Medicine, Indianapolis, IN, USA

## Abstract

**Background:**

The default mode network (DMN) overlaps the brain regions affected by beta‐amyloid (Aβ) deposition during the early Alzheimer's Disease (AD) process. However, limited information is available on the alteration pattern of functional connectivity (FC) related to the DMN according to the stages of AD. This study aims to investigate characteristic FC changes related to the DMN in preclinical AD by comparing them with cognitively normal (CN) individuals with no AD pathology, mild cognitive impaired (MCI) due to AD, and AD dementia.

**Method:**

A total of 396 older adults aged 55 to 88 years, including CN, MCI, and mild AD dementia individuals, were recruited from the Korean Brain Aging Study for the Early Diagnosis and Prediction of Alzheimer's Disease (KBASE) cohort. The participants underwent PiB PET and resting‐state functional MRI. For PET scans, the standard uptake value ratio (SUVR) was used for the analyses; combined regions of the inferior cerebellum and pons were used as the reference region when obtaining SUVRs. DMN FC was computed as the median z‐transformed Pearson correlation for within‐ or between‐network connections using the Schaefer 200 cortical region parcellation. FC within the DMN reflects the FC among regions within the DMN, representing the intrinsic network coherence. FC between the DMN reflects the FC between the DMN and regions outside the DMN, indicating the interaction between the DMN and other brain networks. Pathological Aβ positivity was determined by the presence of significantly high deposition in the posterior cingulate cortex and precuneus. Participants were classified as follows: Aβ‐ CN (CN‐), Aβ+ CN (CN+), Aβ+ MCI (MCI+), and Aβ+ mild AD dementia (AD+) group.

**Result:**

The CN+ group exhibited significantly higher FC within the DMN compared to the other groups, including the CN‐, MCI+, and AD+ groups. The CN+ group also showed significantly lower FC between the DMN than the other groups (Figure).

**Conclusion:**

These findings suggest that increased FC within the DMN and decreased FC between the DMN are characteristic features of preclinical AD. While further studies are needed, the characteristic features of DMN FC in preclinical AD may result from compensatory network activation in response to early Aβ deposition.